# Potential Determinants and Effects of Exclusive Breastfeeding Among Infants at a Tertiary Care Center, Kerala, India

**DOI:** 10.7759/cureus.23185

**Published:** 2022-03-15

**Authors:** Reny Joseph, Jijo J John, Alice David, Lakshmy Sankar, Dary Darvin, Mohammed Yashik

**Affiliations:** 1 Pediatrics, MGM Muthoot Hospital, Kozhencherry, IND; 2 Pediatrics, Believer's Church Medical College Hospital, Thiruvalla, IND; 3 Epidemiology and Public Health, Believer's Church Medical College Hospital, Thiruvalla, IND

**Keywords:** formula feeding, exclusive breastfeeding, infant and young child feeding, child nutrition, breastfeeding

## Abstract

Background: Exclusive breastfeeding (EBF) is the first fundamental right of the child. Globally less than half of the infants are optimally breastfed. Suboptimal breastfeeding can lead to increased respiratory and gastrointestinal infections. This study was undertaken to assess the potential determinants and effects of EBF among infants at a tertiary care hospital in south India since interventions to improve breastfeeding in communities have to be tailored to the needs of the population.

Methods: This cross-sectional study was done among infants at the pediatric unit of a tertiary care hospital in central Kerala, from October 2019 to April 2020, using a structured questionnaire.

Results: Two hundred fifty-seven infants were included in the final analysis. 70.4% of babies were exclusively breastfed for the first six months, although 80.9% were breastfed within the first hour after birth. Among determinants of EBF, unemployed mothers and mothers without a post-graduate degree were more likely to continue EBF for six months (OR 2.8 95% CI [1.6-4.9] and OR 2.7 95% CI [1.5-4.9], respectively). Antenatal counseling appeared to have some beneficial effects but the result was not statistically significant. The mean number of respiratory infections, infections requiring hospitalization, and mean antibiotic use was lower in the exclusively breastfed group, though this result was not statistically significant. However, a significantly lower number of breastfed babies had constipation (OR 0.4, 95% CI 0.2-0.9) when compared to formula-fed babies.

Conclusion: A higher percentage of infants presenting to our hospital has been exclusively breastfed as compared to the state average. Potential determinants of EBF include maternal education and employment and the potential effect of EBF includes protection against constipation. Further emphasis on counseling mothers antenatally, providing postnatal lactation support and counseling, providing mothers with adequate maternity leave will play a major role in promoting EBF in our community.

## Introduction

Breastfeeding is a fundamental human right of every mother and child. It significantly affects child mortality and morbidity. In mothers, lack of breastfeeding is associated with an increase in the incidence of ovarian cancer, premenopausal breast cancer, type 2 diabetes, and metabolic syndrome [1]. The World Health Organization (WHO) recommends initiation of breast milk within the first hour of life, continuing exclusive breastfeeding (EBF) with no other food or drink till six months followed by appropriate complementary food with continued breastfeeding up to the age of two years. According to the WHO, over 800,000 under-five children’s lives could be saved if optimal breastfeeding practices were followed; however, only 44% of infants under six months of age are exclusively breastfed globally [2].

According to India’s National Family Health Survey 5 (NFHS-5) (2019-2020), 66.7% of children in Kerala received breastfeeding within an hour of birth; however, only 55.5% were exclusively breastfed for six months [3]. Though these figures are marginally better than the previous survey in 2015-2016 (64% and 53% respectively), the fact remains that only about 50% of infants receive optimal breastfeeding.

This study was undertaken to understand breastfeeding practices in terms of breastfeeding indicators identified by WHO among infants admitted to a tertiary care hospital in Kerala, South India.

The preprint version was published by Research Square; November 29, 2021, with the following DOI https://doi.org/10.21203/rs.3.rs-1068158/v1.

## Materials and methods

Study design and setting

This study was conducted at a tertiary care facility in central Kerala with about 100 deliveries a month. Antenatal and postnatal lactation counseling sessions were provided by a certified lactational consultant. This was a cross-sectional study of all infants from six months to one year of age who were registered with the hospital and visiting any of the pediatric facilities such as OPD, immunization clinic, emergency room, in-patient wards between October 2019 and April 2020. Following discharge, mothers were interviewed over the telephone using a structured questionnaire by a medical student, who was trained by a consultant pediatrician before the interview. The questionnaire was based on previous similar studies [4,5], and a pilot study was initially conducted among 100 babies born in this hospital, and the questionnaire was modified accordingly.

Exclusion criteria

If there was no response after three calls to contact each phone number provided, children were excluded from the study.

Sample size calculation

Based on the NFHS-5 data, using a prevalence of 55.5% breastfeeding in Kerala, the minimum detectable odds ratio of 2.5, power of 80%, and alpha error of 5% the minimum required sample size is 216 in the ratio of 1:2, where the number of EBF is to be 72 and others to be 144. While NFHS samples included women from all strata of society, our hospital is a private medical college located in one of the richest districts in Kerala. Hence, the population catered to, in our hospital is not representative of the state. Our objective was to tease out potential determinants of EBF in this community which could be the same in other communities of Kerala to varying degrees, rather than estimating the prevalence of the burden.

Study measures

We defined EBF as being fed only breast milk without other formula supplementation for the first six months, as per the recommendation by WHO. Socio-demographic characteristics of the mother include age at delivery, education, employment, gravidity, and type of family. Child characteristics assessed were birth weight, gestation at birth, mode of delivery, and gender. Reasons for discontinuing EBF were noted in detail. To assess the effects of EBF on the infants’ health, occurrences of respiratory, gastrointestinal, and ear infections, requiring antibiotics or hospitalization were noted. Occurrences of constipation were also noted.

Statistical analysis

Descriptive statistics include frequencies and proportions for categorical data and mean and standard deviation for continuous data. Potential determinants and effects of EBF were obtained using z-test, Student t-test, and odds ratio with 95% Confidence Interval. Only variables found to be significant were included in the multivariate logistic regression.

## Results

A total of 2,426 children visited the pediatric facility from October 2019 to April 2020, of which 603 were infants between the ages of six months to one year. Three hundred and forty-six infants were excluded (238 wrong phone numbers, 96 migrated abroad, two deaths, 10 missing data). After exclusion, 257 mother-infant dyads were included in the final analysis. Two hundred and eight babies (80.9%) were breastfed one hour after birth. However, only 181 (70.4%) mothers exclusively breastfed for the first six months of whom 32 (17.7%) did not breastfeed within the first hour. Since we had a high non-response rate (57.4%), the proportion of EBF may not reflect the true value. A sensitivity analysis assuming all 346 non-responders to have EBF, the proportion would be as high as 87.4%, and assuming that none of them EBF, the proportion would be only 30.0%. The midpoint between 30.0% and 87.4% is 58.7%. This is close to but lower than the national prevalence. However, the lowest possible proportion in our hospital is higher than the only other study done in Kerala that we found.

Potential determinants of EBF by univariate analysis are given in Table 1. The average age of the mothers was 29.1 years, the range being 19-42 years. There was no sufficient evidence to show that the age of the mother at delivery had any influence on her choice to exclusively breastfeed her baby (OR 1.2, 95% CI 0.6-2.4, p=0.6). A majority (61.1%) of the mothers had either a professional qualification or a post-graduate degree. 10% of mothers had a high school education, while 29% had a college graduate degree or diploma. Mothers with the education of graduate degree or less were more likely to breastfeed exclusively (OR 2.7, 95% CI 1.5-4.9, p=0.001). About a third (32.2%) of the mothers were employed at the time of pregnancy. Unemployed mothers were significantly more likely to practice EBF (OR 2.8, 95% CI 1.6-4.9, p=0.001). Of the mothers who were employed, there were 19 (25%) mothers who had shorter maternity leaves and needed to return to work before six months post-partum.

**Table 1:** Potential determinants of exclusive breastfeeding by univariate analysis *significant

Determinants	Total, n (%)	Exclusively Breastfed (N=181) n(%)	Not Exclusively Breastfed (N=76) n(%)	P-value	OR (95% CI)
Mother's Characteristics					
Mother’s education: graduate or less	100 (38.9)	82 (45.3)	18 (23.7)	0.001	2.7 (1.5 - 4.9)*
Mother unemployed	174 (67.7)	135 (74.6)	39 (51.3)	0.001	2.8 (1.6 - 4.9)*
Nuclear family	131 (51.0)	93 (51.4)	38 (50.0)	0.8	1.1 (0.6 - 1.8)
Child's Characteristics					
Male Child	127 (49.4)	920(50.8)	35 (46.1)	0.7	1.2 (0.7 - 2.1)
Term Birth	226 (87.9)	160 (88.4)	66 (86.8)	0.8	1.2 (0.5 - 2.6)
Birth weight > 2.5 kg	222 (86.3)	158 (87.3)	64 (84.2)	0.5	1.3 (0.6 - 2.7)
Birth order: > 1	113 (44.0)	83 (45.9)	30 (39.5)	1.0	1.3 (0.8 - 2.2)
Breastfeeding Indicators					
Breastfed within 1 hour of birth	208 (80.9)	149 (82.3)	59 (77.6)	0.3	1.3 (0.7 - 2.6)
Antenatal Counselling Received	233 (90.7)	166 (91.7)	67 (88.2)	0.9	1.5 (0.6 - 3.2)

In the present study, there was not enough evidence to show a gender-based difference in the rates of EBF (OR 1.2, 95% CI 0.7-2.1, p=0.7). Also, there was no sufficient evidence to show a significant difference in the rates of EBF by gestational age at delivery (OR 1.2, 95% CI 0.5-2.6, p=0.8), birth weight (OR 1.3, 95% CI 0.6-2.7, p= 0.5) or birth order (OR 1.3, 95% CI 0.8-2.2, p=1).

The majority (90.7%) of the mothers were given lactation counseling antenatally, which appeared to have a beneficial effect on EBF; however, there was not enough evidence to prove this association (OR 1.5, 95% CI 0.6-3.6, p=0.9). Similarly, most mothers, (80.9%) breastfed within one hour. However, the evidence to establish the association of early initiation and EBF was lacking (OR 1.3, 95% CI 0.7-2.6, p=0.9).

The only variables found to be significant after multivariate analysis is shown in Table 2. The probability of EBF by mothers with a college education or less (OR 2.3, 95% CI 1.3-4.3, p=0.007) and mothers who are unemployed (OR 2.5, 95% CI 1.4-4.9, p=0.002) were highly significant. The discriminatory power of this model is 67.2% (see Table 2).

**Table 2:** Potential determinants of exclusive breastfeeding by multivariate analysis

Determinants	Total, n (%)	OR (95% CI)	P-value	Adjusted OR (95% CI)	P-value
Mother’s education: graduate or less	100 (38.9)	2.7 (1.5 - 4.9)	0.001	2.3 (1.3 - 4.3)	0.007*
Mother unemployed	174 (67.7)	2.8 (1.6 - 4.9)	0.001	2.5 (1.4 - 4.9)	0.002*

**Figure 1:**
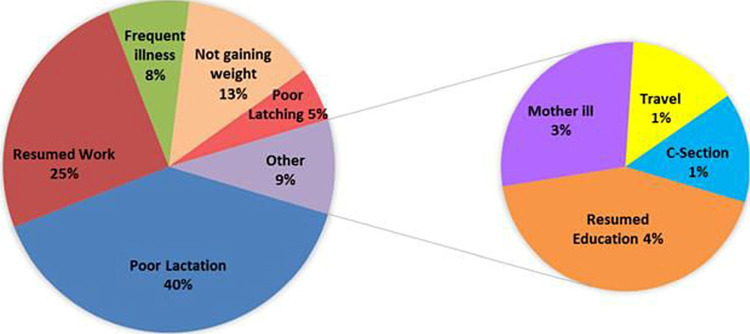
Reasons for not exclusively breastfeeding

The reasons for supplemental feeding are given in Figure 1. Predominant reasons for supplemental feeding were perceived breast milk insufficiency (39.3%), mother resuming work or studies (26.3%), post-infection (either in the mother or child - 13.2%), and poor weight gain (9.2%).

We also examined the effect of EBF on the health of the infant as shown in Table 3. Breastfed babies were less likely to have constipation when compared to non-breastfed babies (OR 0.4, 95% CI 0.2-0.9). However, there was not enough evidence to show that the two groups differed for other conditions such as the number of episodes for respiratory or gastrointestinal infections, need for antibiotics, or need for hospitalization.

**Table 3:** Potential effects of exclusive breastfeeding *significant

Effects	Total	Exclusively Breastfed (N=181)	Not Exclusively Breastfed (N=76)	P-value	OR (95% CI)
	n (%)	n (%)	n (%)		
Infection					
Respiratory	53 (20.6)	37 (20.4)	16 (21.1)	0.6	1.0 (0.5 - 1.9)
Gastrointestinal	5 (1.9)	3 (1.7)	2 (2.6)	0.1	0.6 (0.1 - 3.8)
Hospitalization					
Respiratory	40 (15.6)	28 (15.5)	12 (15.8)	0.9	1.0 (0.5 - 2.0)
Gastrointestinal	15 (5.8)	12 (7.2)	3 (2.6)	0.3	1.8 (0.7 – 1.3)
Antibiotic Used	24 (9.3)	14 (7.7)	10 (13.2)	0.1	0.6 (0.2 - 1.3)
Constipation	42 (16.3)	23 (12.7)	19 (25.0)	0.02	(0.2 - 0.9)*

## Discussion

Breastfeeding is a vital part of providing every child with the healthiest start in life. In a systematic review of published articles from 42 countries, the single intervention that was found to be effective with sufficient evidence, to prevent under-five mortality in low-income countries was breastfeeding. It could have prevented 13% of under-five deaths [6].

Despite extensive efforts by governments and global organizations, the improvements in breastfeeding trends are, at best, modest [7]. As per WHO fact sheets, only 44% of infants are exclusively breastfed till six months globally [2]. In the recently concluded NFHS-5 from India, 12 states and union territories showed a decline in early initiation of breastfeeding, while seven states showed a drop in the EBF rates. In Kerala, 66.7% of babies were breastfed within one hour of birth, while 55.5% received EBF till six months of age [3]. In our study, we found 80.9% of mothers to have breastfed within one hour after birth, and EBF for six months was practiced by 70.4%. In a previous study from Kerala conducted during 2012-2013, Raveendran et al, found the prevalence of EBF to be much lower (21.9%) [6]. However, our study shows a much higher prevalence of breastfeeding (70.4%). Factors that could be attributed to this increase are discussed below.

In low-income countries, numerous factors affect breastfeeding practices such as maternal age, maternal education, maternal employment, cultural and religious practices, living arrangements, antenatal counseling regarding breastfeeding, and professional support at the time of delivery [6,8-10]. Our study also explored all the above factors except cultural and religious practices. Characteristics related to the child such as gender, gestational age, birth weight, and birth order were also considered. Interestingly, only maternal employment and maternal education were found to be significant although the sample size required to examine the above list was adequate.

Mothers who were employed were almost three times more likely to resort to the formula than mothers who were not employed. This finding is similar to a study done in Somali [10]. Moreover, a systematic review of qualitative studies from Sub-Saharan Africa also identified maternal employment as the major barrier to EBF [11]. One of the reasons could be inadequate maternity leave. Various other studies from around the globe have demonstrated a positive correlation between longer maternity leave and EBF rates [12-16]. According to the new Maternity Benefit (Amendment) Act 2017 in India, women are entitled to 26 weeks of maternity leave with full pay, on completion of at least 80 days in an establishment in the 12 months before her expected date of delivery [16]. However, these laws are not uniformly followed. In our study, 19 (25%) mothers who stopped breastfeeding early did so to resume work. On average, they had only 3.6 months of maternity leave. To ensure EBF these laws have to be strictly enforced and variable options including frozen breastmilk should be encouraged [17].

The other factor that affected EBF was maternal education. We found that women with a graduate degree or less were more likely to exclusively breastfeed their babies. A similar paradox was found by Neves et al. in their recently conducted systematic review which was conducted in 81 low and middle-income countries over the past two decades, where they found lower formal maternal education to be positively associated with breastfeeding. They also found the use of a formula to be more among women with the highest education levels [18]. Education and employment status are highly correlated. However, our multivariate analysis showed that the two factors were independently associated with EBF. This implies that irrespective of their education, being employed adversely affects EBF. Similarly, irrespective of their employment status, being highly educated adversely affects EBF. The final model included only maternal education and maternal employment. Together the power to discriminate between those who exclusively breastfed and those who did not is 67% based on c-statistic. This implies that although the above two variables together clearly discriminate, there are other unexplained factors such as postnatal lactation support, that affect this behavior, which we have not studied but discussed below.

Additional support such as antenatal counseling could have made a difference. In our study as the majority of the mothers received antenatal counseling, we could not prove a statistical significance with breastfeeding and antenatal counseling. A randomized control trial from Pakistan showed that mothers who were counseled antenatally were significantly more likely to initiate breastfeeding early and continue EBF for six months [19]. Similarly, the systematic review from Sub-Saharan Africa demonstrated that knowledge of the benefits of EBF was the most common facilitator towards EBF [11]. Therefore, other factors such as postnatal lactation support may play a role. For example, Lin-Lin Su and associates found antenatal counseling and postnatal lactation support to independently improve EBF among mothers in Singapore [20]. In our study, the most common reason given for not continuing breastfeeding was apparent poor lactation (39.4%). Therefore, additional postnatal support for lactation is essential, as it would have resolved many of the other reasons for top feeds.

EBF for six months has many benefits for the mother and baby. Various bioactive factors in breast milk protect the baby against gastrointestinal and respiratory infections. The Generation R Study, a Dutch population-based cohort study found breastfeeding for six months or longer to be protective against lower respiratory tract infections till four years of age [21]. The TEDDY group found breastfeeding for 3-6 months to be protective against respiratory infections, infective gastrointestinal infections, and otitis media [22]. In our study, the mean number of respiratory infections and infections requiring hospitalization was lower in the exclusively breastfed group. Similarly, the mean antibiotic use was also lower in the breastfed group. However, both these results were not statistically significant. This may be because only a small number of children required hospitalization or antibiotics. We did however find a significant association between formula feeding and constipation. Similar results were demonstrated in a multicentric cross-sectional trial among European infants, where formula-fed infants were noted to have more constipation, among other functional gastrointestinal disorders, as compared to breastfed infants [23].

Global breastfeeding collective is a partnership of prominent international organizations including UNICEF and WHO, to improve breastfeeding trends across the globe. There are seven steps recommended, which, if implemented at all levels of the community and government, will ensure an increase in breastfeeding rates [24].

EBF for the first six months of life requires the collective effort of the community in the form of antenatal counseling, postnatal lactation support, family support, employee benefits, and paid maternity leave. The beneficial effects of EBF extend beyond the mother-infant dyad. It leads to healthier communities and improves the economy of the country. 

Limitations

Although we had an adequate sample size to determine the factors associated with EBF, both potential determinants, and potential effects, the large nonresponse rate could bias the results if those who responded were all of a particular characteristic. Recall bias too could play a role in determining the potential effects of EBF. Also, the sample group studied was of a particular demographic profile, and larger studies with a more diverse population are required to identify the determinants of breastfeeding across the state.

## Conclusions

A significantly high proportion of infants in our study group has been exclusively breastfed. The major determinant of EBF was maternal education and employment. Children who were not exclusively breastfed had a higher number of gastrointestinal and respiratory infections and had a significant association with constipation. Interventions tailored to the needs of the population being targeted to improve breastfeeding rates such as counseling mothers antenatally, providing postnatal lactation support and counseling, providing mothers with adequate maternity leave will play a major role in promoting EBF.
